# Novel Insights on the Use of L-Asparaginase as an Efficient and Safe Anti-Cancer Therapy

**DOI:** 10.3390/cancers14040902

**Published:** 2022-02-11

**Authors:** Maaike Van Trimpont, Evelien Peeters, Yanti De Visser, Amanda M. Schalk, Veerle Mondelaers, Barbara De Moerloose, Arnon Lavie, Tim Lammens, Steven Goossens, Pieter Van Vlierberghe

**Affiliations:** 1 Cancer Research Institute Ghent (CRIG), 9000 Ghent, Belgium; maaike.vantrimpont@ugent.be (M.V.T.); evelien.peeters@ugent.be (E.P.); yanti.devisser@kuleuven.be (Y.D.V.); barbara.demoerloose@uzgent.be (B.D.M.); tim.lammens@ugent.be (T.L.); steven.goossens@ugent.be (S.G.); 2 Department of Biomolecular Medicine, Ghent University, 9000 Ghent, Belgium; 3 Department of Diagnostic Sciences, Ghent University, 9000 Ghent, Belgium; 4 Department of Biomedical Molecular Biology, Ghent University, 9000 Ghent, Belgium; 5 Department of Imaging and Pathology, KU Leuven, 3000 Leuven, Belgium; 6 Department of Biochemistry and Molecular Genetics, University of Illinois, Chicago, IL 60607, USA; aschalk@uic.edu (A.M.S.); lavie@uic.edu (A.L.); 7 Department of Pediatric Hemato-Oncology and Stem Cell Transplantation, Ghent University Hospital, 9000 Ghent, Belgium; veerle.mondelaers@uzgent.be; 8 Department of Internal Medicine and Pediatrics, Ghent University, 9000 Ghent, Belgium; 9 The Jesse Brown VA Medical Center, Chicago, IL 60607, USA

**Keywords:** asparaginase, asparagine, glutamine, acute lymphoblastic leukemia, solid cancers

## Abstract

**Simple Summary:**

L-asparaginase (L-ASNase) therapy is key for achieving the very high cure rate of pediatric acute lymphoblastic leukemia (ALL), yet its use is mostly confined to this indication. One main reason preventing the expansion of today’s FDA-approved L-ASNases to solid cancers is their high toxicity and side effects, which become especially challenging in adult patients. The design of optimized L-ASNase molecules provides opportunities to overcome these unwanted toxicities. An additional challenge to broader application of L-ASNases is how cells can counter the pharmacological effect of this drug and the identification of L-ASNases resistance mechanisms. In this review, we discuss recent insights into L-ASNase adverse effects, resistance mechanisms, and how novel L-ASNase variants and drug combinations can expand its clinical applicability, with a focus on both hematological and solid tumors.

**Abstract:**

L-Asparaginase (L-ASNase) is an enzyme that hydrolyses the amino acid asparagine into aspartic acid and ammonia. Systemic administration of bacterial L-ASNase is successfully used to lower the bioavailability of this non-essential amino acid and to eradicate rapidly proliferating cancer cells with a high demand for exogenous asparagine. Currently, it is a cornerstone drug in the treatment of the most common pediatric cancer, acute lymphoblastic leukemia (ALL). Since these lymphoblasts lack the expression of asparagine synthetase (ASNS), these cells depend on the uptake of extracellular asparagine for survival. Interestingly, recent reports have illustrated that L-ASNase may also have clinical potential for the treatment of other aggressive subtypes of hematological or solid cancers. However, immunogenic and other severe adverse side effects limit optimal clinical use and often lead to treatment discontinuation. The design of optimized and novel L-ASNase formulations provides opportunities to overcome these limitations. In addition, identification of multiple L-ASNase resistance mechanisms, including ASNS promoter reactivation and desensitization, has fueled research into promising novel drug combinations to overcome chemoresistance. In this review, we discuss recent insights into L-ASNase adverse effects, resistance both in hematological and solid tumors, and how novel L-ASNase variants and drug combinations can expand its clinical applicability.

## 1. Introduction

Asparagine (Asn) is a non-essential amino acid (AA), taken up via the diet or, alternatively, synthesized from central metabolic pathways in which Asn is produced from aspartic acid (Asp) and glutamine (Gln) by the enzyme asparagine synthetase (ASNS) [1,2,3,4]. L-asparaginase (L-ASNase) was first described in 1904 as a non-human enzyme that hydrolyses Asn into Asp and ammonia in different bovine tissues [5]. In 1953, Kidd et al. reported that the serum from guinea pigs could inhibit the growth of transplanted lymphomas in mice, but it took almost another 10 years before Broome et al. pinpointed that this inhibition was due to its L-ASNase enzyme activity [6,7]. In 1966, the first use of purified guinea pig serum to treat a boy with acute lymphoblastic leukemia (ALL) was reported [8]. As guinea pig serum is an expensive and limited resource for L-ASNase, the pharmaceutical industry rapidly moved towards alternative L-ASNases from bacterial sources for large-scale production of sufficient amounts of L-ASNase for cancer treatment purposes [9,10].

**Figure 1:**
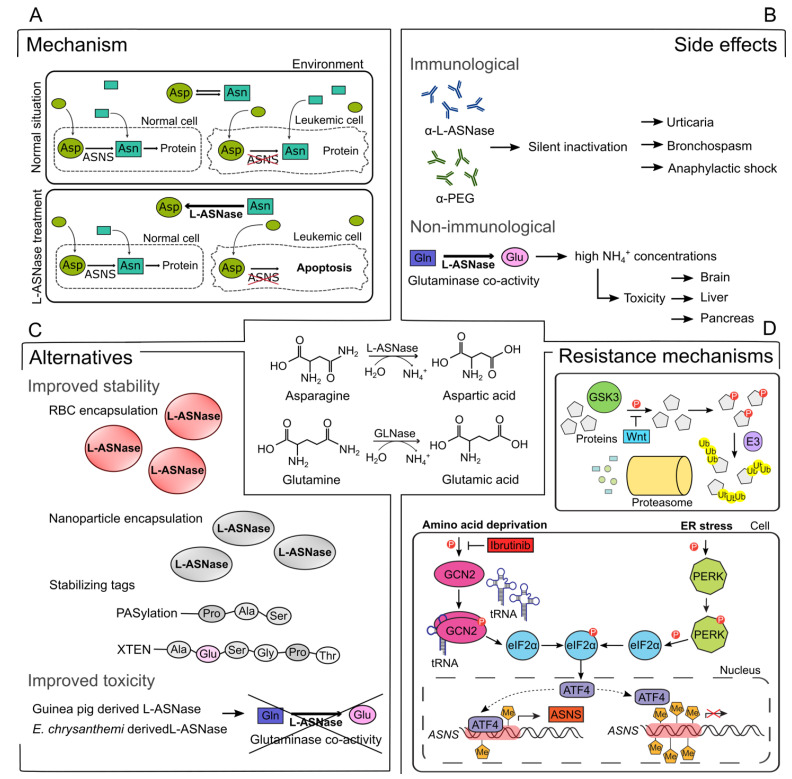
Overview of L-asparaginase (L-ASNase) mechanism, side effects, alternatives, and combination therapies. (**A**) Mechanism of L-ASNase treatment in leukemic cells. Administration of L-ASNase depletes Asn from the environment, resulting in selective apoptosis of leukemic cells due to the lack of ASNS. Asn, asparagine; Asp, aspartic acid; ASNS, asparagine synthetase; L-ASNase, L-asparaginase (**B**) Immunological and non-immunological side effects related to ASNase treatment. Production of α-L-ASNase or α-PEG antibodies causes silent inactivation, which results in multiple side effects. The glutaminase co-activity of L-ASNase gives rise to a high concentration of NH_4_^+^, which are correlated with brain, liver, and pancreas toxicities. Gln, glutamine; Glu, glutamic acid, PEG, monomethoxypolyethylene glycol; GLNase, glutaminase (**C**) Multiple stabilizing strategies for ASNase based on encapsulation or tag addition that are under investigation or already approved in the clinic. RBC, red blood cell; Pro, proline; Ala, alanine; Ser, serine; Gly, glycine, Thr, Threonine (**D**) GSK3a-dependent protein ubiquitination and degradation and the GCN2-ATF4-ASNS axis are affected by ASNase treatment and have the ability to cause resistance. ER, endoplasmic reticulum; GSK3, glycogen synthase kinase-3; Wnt, Wingless/Integrated; E3, E3 ubiquitin ligase; Ub, ubiquitin; GCN2, general control nonderepressible 2; tRNA, transfer RNA; elF2α, eukaryotic translation initiation factor 2α; PERK, protein kinase RNA-like ER kinase; ATF4, activating transcription factor 4; Me, methyl.

Currently, L-ASNase is mainly used as a cornerstone drug in the therapeutic regimen for childhood ALL. In contrast to healthy cells, ALL lymphoblasts largely lack ASNS expression, and thus depend on an extracellular source of Asn for their survival [11,12,13,14,15]. As large amounts of Asn are indeed necessary for ALL cell growth, depleting circulating Asn by L-ASNase administration results in starvation and selective apoptosis of the leukemic blasts (Figure 1A). More recently, accumulating evidence suggests that L-ASNase could also have clinical potential for the treatment of other subtypes of leukemia or certain aggressive solid tumors [16,17,18,19,20]. Nevertheless, use of this drug in adult cancer populations has largely been hampered by its toxicity profile, which includes severe immunological side effects as well as non-immune related toxicities such as pancreatitis, liver toxicities, coagulopathy, and neurotoxicity [21,22].

## 2. Bacterial L-ASNase Used in the Clinic for ALL Therapy

Five different L-ASNase preparations are currently used in the clinic. Two variants, Erwinase^®^ and Rylaze^®^, are both of the same *Erwinia chrysanthemi* ansB gene product but differ in their bio-manufacturing process [23,24]. The three others are derived from the *Escherichia coli* Type 2 L-ASNase. One is a native formulation (brand names Elspar^®^; Kidrolase^®^; Spectrila^®^; and others), while the two others are stabilized formulations, resulting from the covalent conjugation of monomethoxypolyethylene glycol (PEG) to lysine resides on the enzyme via a succinimidyl succinate linker (Oncaspar^®^) [25,26] or a succinimidyl carbonate linker (Asparlas^®^) [27,28]. The addition of the PEG-tag delays elimination from the body and increases the half-life of PEG-L-ASNase in humans towards 5.5 days (Oncaspar^®^) [29,30,31] and 16.1 days (Asparlas^®^) [28]. Given its enhanced stability and earlier development, Oncaspar^®^ is the main enzymatic variant that is currently used in the clinic as front-line therapy for the treatment of childhood ALL [32,33,34]. An overview of all the above discussed L-ASNase variants is provided in Table 1.

In addition to their L-ASNase activity, all clinically approved bacterial L-ASNases also hydrolyze Gln into glutamic acid (Glu) and ammonia. The clinical relevance of this glutaminase co-activity in the context of ALL remains largely unclear, with conflicting reports in the literature about its putative antileukemic effect [38,39,40,41]. First, it was shown in vitro that this glutaminase co-activity was not required for its anticancer effect against *ASNS* negative leukemic cells [38]. In line with this notion, Nguyen et al. indeed showed that glutaminase co-activity was not essential for inducing an in vivo L-ASNase effect in a xenograft model of *ASNS*-negative SUP-B15 leukemia cells [40]. However, more recently, it was suggested that a durable and long-lasting anticancer effect in the same SUP-B15 xenograft model could only be obtained using an L-ASNase variant that retained its glutaminase co-activity [41]. Therefore, in the future, additional studies will be required to unambiguously show if glutaminase co-activity is truly required for a long-term antileukemic response in *ASNS*-negative tumor cells or not.

### 2.1. Immunological Side Effects

Due to its bacterial nature, immune responses to L-ASNase during treatment can occur in 30–70% of patients and have been associated with the generation of potentially neutralizing antibodies [30]. Clinical hypersensitivity reactions range from light local symptoms (urticaria) to systemic reactions (bronchospasm and anaphylactic shock) [30,42] (Figure 1B). The L-ASNase preparation, intensity, and consistency of dosing, as well as the administration route can all influence the likelihood of developing an immune reaction during L-ASNase therapy [34,43,44]. For example, it was shown that native *E. coli* L-ASNase is more immunogenic than the PEG-L-ASNase [45,46,47,48]. The antibodies generated during these hypersensitivity reactions can result in increased L-ASNase clearance and, therefore, a reduction or neutralization of the catalytic activity of L-ASNase [49,50]. In addition, circulating antibodies can also develop in the absence of any obvious clinical signs of allergy, a phenomenon known as silent inactivation, which can often go undetected [34,42,51]. However, recent work described that ALL patients treated with PEG-L-ASNase can also develop antibodies against the PEG-tag itself, thereby limiting tolerance and efficacy [52,53]. For this reason, additional research on more immunotolerant tags is currently ongoing, including XTEN, a non-immunogenic and biodegradable unstructured polymer [54] as well as PASylation [31,55,56] and red blood cell (RBC) encapsulation [57,58] (Figure 1C). Although the importance of switching from *E. coli* PEG-L-ASNase to *E. chrysanthemi* L-ASNase during ALL therapy after silent inactivation has been clearly established by several reports [49,59,60], hypersensitivity towards the *Erwinia* derived enzyme can also still occur in 3–33% of patients [23,42,61,62]. Albeit premedication with steroids or antihistamines is known to reduce clinical hypersensitivity symptoms, it may not prevent the development of antibodies [48]. Therefore, immunological responses against clinically used bacterial L-ASNase formulations can lead to early termination of treatment and decreased prognosis [50,63,64,65]. The active search towards and development of alternative L-ASNases aims to eliminate these problems by creating additional treatment options.

The efficiency of Asn depletion can be determined by serum Asn concentration, the presence of PEG-L-ASNase antibodies, and L-ASNase activity. However, due to the lack of tests for anti-L-ASNase antibodies that distinguish between inactivating or non-inactivating antibodies and the fast ex vivo metabolism of Asn in the presence of L-ASNase, the evaluation of L-ASNase activity remains the only option to monitor silent inactivation in patients during therapy [34,42,66]. Indeed, real-time monitoring of L-ASNase activity during treatment regimens is critical to avoid unfavorable outcomes in pediatric ALL due to suboptimal L-ASNase therapy, as recently shown in the context of ALL patients treated according to the Nordic Society of Pediatric Hematology and Oncology (NOPHO) ALL2008 protocol [67].

Of note, in recent years the drug supply of *E. chrysanthemi* L-ASNase was often limited due to manufacturing issues, thereby potentially harming optimal treatment outcome for hypersensitive ALL patients. To address this, recombinant DNA technology has recently been explored to increase L-ASNase production yield and overcome problems associated with drug shortage. Indeed, a new recombinant version of *E. chrysanthemi* L-ASNase, Rylaze^®^, also known as JZP-458, has been developed and taken forward into a clinical trial (NCT04145531). Recently, it has been approved by the FDA as part of a treatment regimen for children and adults with ALL or lymphoblastic lymphoma [24,68,69]. Rylaze^®^ will help alleviate the shortage of additional L-ASNase for the treatment of ALL patients that develop hypersensitivity against PEG-L-ASNase [24].

### 2.2. Non-Immunological Side Effects

Finally, non-immune related toxicities that have been associated with L-ASNase administration occur with lower frequency but can develop with higher severity to affect treatment outcomes and include pancreatitis, liver toxicity, coagulopathy, and neurotoxicity [21,30,70,71] (Figure 1B). These toxicities are, at least partially, caused by residual glutaminase co-activity that is present in all clinically used L-ASNases [72,73,74,75,76]. This co-activity causes hydrolyzation of Gln into Glu and ammonia. The extensive ammonia production might be an important factor that contributes to L-ASNase-induced liver dysfunction and neurotoxicity [74,77,78,79]. It remains to be established whether this glutaminase co-activity is truly required for a long-term antileukemic response in *ASNS*-negative tumor cells [38,39,40,41]. Therefore, reducing the glutaminase co-activity of available L-ASNase may be advantageous to lessen toxic side effects, enabling patients to complete their treatments and, improve their outcomes as well as be used more frequently in the treatment of adults with ALL.

Of note, besides L-glutamine, also asparaginyl-transfer RNA has been reported to be a substrate for L-ASNase [80]. On top, some L-ASNases may have alternative activities that putatively play a partial role in their therapeutic activity and associated adverse side-effect. *E. coli* L-ASNase has been demonstrated to bind and inhibit the concanavalin A receptor [81,82]. More recently, an asparagine-independent cytotoxicity mechanism was reported specifically for a *Rodosprillum rubrum* mutant L-ASNase [83]. In contrast to other bacterial L-ASNases, this RrA variant can penetrate cancer cells via a clathrin receptor-dependent manner resulting in suppression of telomerase activity and asparagine-independent cytotoxicity [83,84].

## 3. ASNS Promoter Methylation as Biomarker for L-ASNase Sensitivity in ALL

Human ASNS activity is highly regulated at the transcriptional level via two main signaling pathways which are switched on after cellular stress to ensure survival [4]. The amino acid response (AAR) pathway is activated in response to intracellular AA depletion and an imbalance in AA homeostasis [85,86]. AAs are required for the synthesis of many proteins, lipids or nucleic acids and serve as essential nutrients for rapidly proliferating cancer cells [87,88]. GCN2 is a serine/threonine-protein kinase that senses AA deficiency, through binding with uncharged transfer RNA (tRNA), thereby functioning as a key regulator of the AAR [89,90,91,92,93]. Likewise, endoplasmic reticulum stress can also increase *ASNS* transcription via the activation of PERK kinases in the unfolded protein response (UPR) pathway [94]. The activation of both GCN2 and PERK kinases leads to the phosphorylation of the eukaryotic initiator factor eIF2α, which increases the levels of the transcription factor ATF4 [95,96,97]. If the promoter region of *ASNS* is hypomethylated, ATF4 can bind and cause stress-induced *ASNS* upregulation. In contrast, when the *ASNS* promoter is hypermethylated, no ATF4-induced ASNS expression will be observed [98] (Figure 1D).

In line with this notion, Jiang et al. recently showed that DNA hypermethylation at the *ASNS* promoter prevents its transcriptional expression following AA depletion in ALL cells, potentially explaining why most ALL cells are so exquisitely sensitive to this AA depletion therapy [99]. Furthermore, Touzart et al. also recently showed that the *ASNS* promoter methylation status strongly correlates with L-ASNase sensitivity in T-ALL cell lines and patient derived xenografts [100]. Therefore, although it was initially thought that low or absent ASNS expression would determine L-ASNase sensitivity in ALL [101,102], these more recent studies now suggest that *ASNS* promoter methylation in combination with low ASNS expression at diagnosis most likely serves as a better biomarker to predict L-ASNase sensitivity in this disease [99,100,103,104,105].

## 4. Expanding the Use of L-ASNase Therapy beyond ALL in Children

The observation that more cancer subtypes have lower ASNS expression compared to their normal healthy counterparts also prompted efforts to expand the use of L-ASNase outside the field of pediatric ALL (Table 2).

### 4.1. Acute Monocytic Leukemia

For example, in the context of leukemia, variable ASNS expression has also been observed in AML. In particular acute monocytic leukemia (AML-M5) patients and AML with monosomy of chromosome 7 have been reported to have lower ASNS expression, which was correlated with sensitivity to L-ASNase administration ex vivo [118,119]. Subsequently, in vivo efficacy for L-ASNase in both adult and pediatric AML was seen when combined with methotrexate or a high dose cytarabine [107,109]. However, as the correlation between L-ASNase-sensitivity and ASNS expression in AML is not strong, and bioavailability of Gln has been demonstrated to be important for AML growth, debate is ongoing whether the cytotoxic effects of bacterial L-ASNase on AML cells is also due to their glutaminase co-activity [106,108]. In addition, a recent study also investigated the effects of L-ASNase treatment on the leukemic bone marrow microenvironment and its relationship with L-ASNase sensitivity in AML. More specifically, while L-ASNase had a clear cytotoxic effect on AML blasts, including the leukemic stem cell subpopulation, the effect of the drug was shown to be counteracted by the mesenchymal stromal cells and the monocytes/macrophages within this niche. This protective effect could be either attributed to the release of Asn or due to drug clearance mediated by lysosomal cysteine proteases such as cathepsine B [120,121]. More research will be necessary to further evaluate the role of the tumor microenvironment on L-ASNase efficacy.

### 4.2. Solid Cancers

In relation to non-hematological tumors, tissue microarrays have been used to identify ASNS-free cancer cells within a variety of solid cancer subtypes. It was suggested that ASNS protein levels could be used as a predictive biomarker to estimate susceptibility to L-ASNase treatment [122]. Lorenzi et al. indeed confirmed the potential use of this biomarker in ovarian cancer and demonstrated a strong negative correlation between ASNS expression levels and L-ASNase sensitivity [18]. Furthermore, 15% of tested ovarian cancers in a tissue microarray had low ASNS levels, especially ovarian clear cell carcinomas (OCCC). Based on this, it was hypothesized that patients with advanced OCCC, who are known to have a worse prognosis, could potentially benefit from L-ASNase therapy. As a result, a clinical trial for the use of PEG-L-ASNase in advanced ovarian cancer was initiated. However, the study was prematurely terminated in Phase II due to excessive toxicities observed, including persistent nausea, pancreatitis, and allergic reactions. Similar to AML, the glutaminase co-activity might play an important role in the cytotoxic activity of the bacterial L-ASNases in ovarian cancer. Purwaha et al. reported that cytotoxic effects induced by PEG-L-ASNase were primarily associated with Gln concentrations instead of Asn concentrations in culture conditions of OVCAR-8 cells [110].

The genetic background of tumor cells might also be an important variable that contributes to the susceptibility of tumor types to L-ASNase treatment or other antimetabolic treatments. For example, pancreatic cancer is generally very sensitive to AA depletion. In the majority of pancreatic ductal adenocarcinoma (PDAC) cases, KRAS has been found to be mutationally activated [123]. Continuous KRAS activation alters and regulates Gln metabolism to optimize cancer cell growth [124,125]. Gln is metabolized through upregulation of glutamate-oxaloacetate transaminase 2 (GOT2) into aspartate [126]. As a result, Gln and Asn dependencies are often seen in PDAC, creating a key vulnerability that can be exploited using L-ASNase treatment [111]. Dufour et al. screened 99 human PDAC for ASNS expression levels and showed that 52% of the pancreatic tumors could be classified as ASNS-low tumors as compared to healthy pancreatic tissue, which displayed high ASNS levels [17]. Furthermore, KRAS mutations are also frequently identified in colorectal cancer, suggesting that L-ASNase therapy might also be relevant in a subset of colorectal cancer patients [127]. Indeed, in combination with rapamycin, PEG-L-ASNase significantly suppressed the growth of KRAS-mutant colorectal cancer [112]. In this context, SOX12 was identified as an important regulator of GOT2 and ASNS, and knockdown of either one of these enzymes suppressed SOX12-mediated proliferation and metastasis in colorectal cancer [128]. 

Using metastatic breast carcinoma cell lines, Knott et al. recently demonstrated that Asn bioavailability governs their metastatic potential [20]. In vitro, Asn supplementation in AA deficient culture conditions significantly promoted the invasive properties of these cells. In vivo, *ASNS* silencing or Asn deprivation from food did not affect primary tumor growth but significantly reduced the number and size of metastatic nodules in the lungs [20]. This decrease in metastatic potential was correlated with the absence of Twist, a regulator of epithelial to mesenchymal transitions [20,129]. It was proposed that invasive breast cancer cells, which obtained mesenchymal traits, are more in need of Asn levels compared to their less invasive epithelial counterparts [20,130]. Alternatively, their altered expression profile, e.g., by activation of the WNT/STOP pathway, may force them to become dependent on alternative sources of free Asn [3,131]. On the other hand, supplementation of Asn restored cell proliferation and survival of multiple human breast cancer cell lines grown in Gln-depleted media [132]. Gln is known to be important for primary tumor growth in several breast cancer subtypes [111]. For example, many MYC-expressing Triple Negative Breast Cancer (TNBC) tumors show Gln addiction [133]. It was shown that L-ASNase together with a glutamine synthetase inhibitor may exert a complete inhibition of the Gln metabolism and enhances the anticancer effect in solid tumor types that are highly dependent on Gln including hepatocellular carcinoma and TNBC [134,135,136]. So, L-ASNase variants with glutaminase co-activity may be the most effective for breast cancer patients as they would both suppress growth of Gln-dependent primary tumors as well as Asn-dependent metastasis. However, a five-drug combination was tested for treatment of metastatic breast cancer in a clinical trial in 1980: 5-fluorouracil, adriamycin, cyclophosphamide, methotrexate, and L-ASNase. Addition of L-ASNase and methotrexate led to increased symptoms of stomatitis and increased diarrhea, and the trial had to be terminated due to overall increased toxicity [137]. Therefore, also for breast cancer, L-ASNases with low glutaminase co-activity may allow decreased toxicity and prolonged treatment.

### 4.3. Glioblastoma

ASNS was revealed to be both a tumor growth suppressor and a critical component for metastasis in hepatocellular carcinoma. Low ASNS levels were correlated with a worse prognosis and increased metastatic potential; however, it was correlated with higher sensitivity to L-ASNase both in vitro and in vivo [114]. In addition, Li et al. noticed that a subset of hepatic cancers showed a hypermethylated *ASNS* promoter alongside low ASNS expression [115]. As mentioned above, *ASNS* promoter methylation inhibits the ability to restore ASNS expression and acquire L-ASNase resistance. As such, those hepatocellular carcinoma patients with low ASNS expression and hypermethylation of the *ASNS* promoter may benefit the most from long-term L-ASNase treatment. 

Over the last decade, the use of L-ASNase for the treatment of glioblastoma has also been evaluated. Glioblastoma is associated with very poor survival rates and a lot of research is currently being performed to establish new therapeutic options for these patients [138]. One of the major problems is the presence of the blood-brain barrier (BBB), which makes it difficult for therapeutic agents with molecular weight over 400 Da and low lipid solubility to reach the tumors [139]. However, in the case of L-ASNase treatment, Asn is depleted systemically in the blood and therefore potentially overcomes the challenge of crossing the BBB. Multiple studies in children with ALL have already hypothesized that the loss of Asn in the peripheral blood is followed by a decline of Asn in the cerebrospinal fluid (CSF), but unfortunately the underlying mechanism of this pharmacodynamic effect is not fully understood [140,141,142]. While literature shows that different L-ASNase formulations can deplete Asn levels in the CSF in ALL [143,144,145], the impact on clinical outcomes has not been clear [142,146,147]. It has been reported that insufficient Asn depletion in the CSF may be associated with a higher likelihood of having a central nervous system (CNS) relapse in ALL [73]. Many studies fail to show any measurable L-ASNase enzyme activity in the CSF, although Riccardi et al. hypothesized the presence of activity in the CSF of rhesus monkeys after native L-ASNase administration [148]. More recently, Ballerini et al. showed a complete Asn depletion in serum and CSF in healthy rats with both the native L-ASNase formulations derived from *E. chrysanthemi* and *E. coli*, and to a lesser extent for PEG-L-ASNase. In addition, they demonstrated that L-ASNase enzymatic activity could be measured in the CSF and was more evident for non-PEGylated formulations, which may be explained by the difference in molecular weight between the different L-ASNase preparations [149]. These results indicate that L-ASNase may be beneficial for the treatment of brain tumors, including glioblastoma. 

Panosyan et al. demonstrated that L-ASNase treatment in vitro resulted in a significant growth reduction in multiple glioblastoma cell lines. The authors also showed the possibility of combining L-ASNase with DNA-damaging drugs, such as temozolomide. Experiments on xenograft tumors in vivo revealed potential synergistic effects between L-ASNase and temozolomide treatment [116]. The combination of both drugs attenuated tumor growth significantly more than either the control or the temozolomide monotherapy groups. However, similar to what has been observed for other solid cancers, it remains unclear whether growth inhibition of glioblastoma is a consequence of Asn or Gln deprivation. Ohba et al. demonstrated that the combination of L-ASNase and 6-Diazo-5-oxo-l-norleucine, a Gln analog that binds to the active sites of Gln-utilizing enzymes and inhibits glutaminase and ASNS, induced a synergistic anti-proliferative effect in glioma cells [117]. In this way, L-ASNase resistance via the upregulation of ASNS can be overcome in glioblastoma.

In summary, many hematological and solid cancer types have potential for sensitivity to Asn deprivation. However, further studies are required to better understand this vulnerability at the molecular level. To expand the use of L-ASNase treatment to other neoplasms, a combination of predictive biomarkers will be necessary to identify the subgroups of patients susceptible to this metabolic therapy. More specifically, we believe that low ASNS expression in combination with ASNS promoter hypermethylation would serve as a good predictor for L-ASNase sensitivity.

## 5. The Development of Novel L-ASNase Variants

L-ASNase has clinical potential for the treatment of other cancer subtypes in addition to childhood ALL. However, the clinical utilization of this drug for adult malignancies has not been widely possible due to its high toxicity profile. There is a clear unmet clinical need for alternative L-ASNase formulations that possess fewer non-immune related toxicities while also being less immunogenic [143]. These novel agents would enable long term L-ASNase treatment in adult cancer patients, improving patient outcomes, and should be prioritized for clinical testing in specific adult cancer populations.

### 5.1. L-ASNase Red Blood Cell Encapsulation

Erytech Pharma created an alternative L-ASNase formulation by encapsulating *E. coli* L-ASNase into RBCs (GRASPA^®^), resulting in a significant increase in half-life as RBCs have a lifespan of about 120 days. In addition, this encapsulation also protects the enzyme, resulting in decreased immunogenicity [150,151]. The formulation of GRASPA^®^, initially called “Eryaspase”, has evolved during its development. Two sources of L-ASNase have been used as raw material and are encapsulated in the RBCs: native (Kidrolase^®^) or recombinant L-ASNase (Spectrila^®^). Several completed studies were conducted with Kidrolase^®^ and showed promising results: three studies in ALL-GRASPALL 2005-01, 2009-06, SA2-2008- and one study in pancreatic carcinoma GRASPANC 2008-02 [152,153]. Later, additional trials were performed with Spectrila^®^ in ALL (GRASPALL 2012-09) and pancreatic cancer (GRASPANC 2013-03). However, in 2018, GRASPA^®^ was not approved for the treatment of ALL by the European Medicines Agency (EMA) due to insufficient evidence and data on safety and efficacy. At the time of withdrawal, the EMA concluded that the benefits of GRASPA^®^ did not outweigh its risks [154]. Notably, for pancreatic cancer, this variant successfully underwent Phase 2b clinical trials [155,156] and is currently in Phase 3 trials [157]. It is also in a Phase 2 clinical trial for TNBC (TRYbeCA-2 trial). Very recently, Eryaspase^®^ was granted Fast Track Designation by the FDA in patients who developed hypersensitivity to *E. coli* L-ASNase [158].

### 5.2. L-ASNase Variants with Reduced or Absent Co-Activity

Most non-immune related toxic side effects of L-ASNase therapy have been attributed to its glutaminase co-activity. Therefore, a reduction of glutaminase co-activity might effectively improve the toxicity profile of L-ASNase variants for the treatment of tumors with low or absent ASNS expression combined with *ASNS* promoter methylation [31,36]. As previously discussed in this review, a mutant form of *E. chrysanthemi* L-ASNase has been reported with ultra-low glutaminase co-activity [40]. Due to low toxicity, this variant might be suitable for clinical testing in the adult ALL setting. In addition, it would also be interesting to evaluate the cytotoxic effect of this L-ASNase variant in a variety of non-ALL cancer cell lines.

*Wolinella succinogenes* derived L-ASNase (*WoA*) was initially reported as a glutaminase-free L-ASNase variant. Reinert et al. compared this variant with *E. coli* derived L-ASNase and showed that *WoA* did not suppress the immune response in mice and lacked hepatotoxicity [159,160,161,162]. In addition, it was demonstrated that PEG-*WoA* caused less toxic side effects compared to PEGylated *E. coli* L-ASNase, suggesting that *WoA* would potentially be a safer drug due to its glutaminase-free properties [163]. Unfortunately, in the early 2000s, *WoA* was evaluated clinically through a US National Cancer Institute Rapid Access to Intervention Development (NCI RAID) grant, and was found to be very toxic and unexpectedly had significant L-glutaminase activity [164]. Meanwhile, whole genome sequencing of *W. succinogenes* revealed a serine at position 121 [165], in conflict with a proline at this position as previously reported by Lubkowski et al. [166]. In 2017, it was confirmed by Nguyen et al. that the *WoA* variant with a proline at position 121 (*WoA*-P_121_) had a higher L-glutaminase activity in contrast to the *WoA* variant with serine at position 121 (*WoA*-S_121_), highlighting the critical role of this residue regarding substrate specificity [164]. 

In addition, Reinert et al. also show that Gln is reduced in liver and spleen after a single administration of *E. coli* L-ASNase, which was not observed for *WoA* derived L-ASNase [167]. Remarkably, a significant increase in Glu levels was observed for the *WoA* treated group. The authors suggested that although Gln is not enzymatically cleaved by *WoA*, its turnover in mice is increased in response to Asn depletion, as Gln provides the NH_3_-group required to make more Asn, leading to a greater production of Glu [167]. Interestingly, this impact on Gln levels during L-ASNase treatment is not always clear in the clinic. This is demonstrated by Tong et al. who showed no depletion of Gln during L-ASNase therapy in ALL pediatric patients [49]. Nevertheless, the decrease of Gln levels after L-ASNase therapy was also observed by Emadi et al. who showed that crisantaspase, produced by *E. chrysanthemi*, caused nearly complete Gln depletion in R/R acute myeloid leukemia (AML) patients with no dose-limiting toxicity, while retaining its antileukemic activity [108]. More recently, they also demonstrated that the long-acting PEG-crisantaspase completely depletes Gln serum levels in AML mice [168]. Nikonorova et al. looked at liver function and toxicity after *E. coli* L-ASNase administration in juvenile and adult mice, and found significantly decreased levels of Gln, together with increased Glu levels [169]. Finally, Nguyen et al. clearly show that *E. chrysanthemi* derived L-ASNase significantly reduced the Gln levels in mice 24 h after L-ASNase administration [40].

Another interesting class of L-ASNase variants are derived from guinea pigs [170,171,172,173]. They are completely glutaminase-free and represent the only mammalian L-ASNase possessing clinically relevant kinetic properties [171]. Interestingly, from an immunogenic perspective, this enzyme already shares ~70% of its sequence homology with the largely inactive human enzyme. Unfortunately, the wild-type versions of known human L-asparaginases are not suitable replacements for the clinically used bacterial enzymes since they possess a very high K_M_ (Michaelis constant) value for Asn [9,174,175], which indicates a low affinity for its substrate. Given the physiological concentration of Asn in blood (~50 μM) [4], the enzyme must have an Asn K_M_ in the low micromolar range to be clinically relevant [172]. Recent work showed that further humanization of this guinea pig L-ASNase towards 85% homology has been possible through DNA shuffling without losing its in vitro antileukemic activity [170,171,172]. Therefore, this humanized guinea pig L-ASNase might serve as an optimal candidate for further clinical development as it might combine fewer non-immune related toxicities with less immunogenicity.

### 5.3. Enzyme Engineering and Bioprospecting

Many attempts have been made to improve the characteristics of existing L-ASNase variants through enzyme engineering. Several methods have been employed, examples being site-directed mutagenesis and directed evolution. The main goal is four-fold: the improvement of in vitro and in vivo stability and enzymatic activity and the reduction of immunogenicity and toxicity. A longer half-life can be obtained by modifying protease cleavage sites in the enzyme. Recently, it was shown that an N24S mutation in *E. coli* L-ASNase generates increased protease-resistance and thermal stability [176]. Secondly, as mentioned before, toxicity of L-ASNases can be at least partly contributed to glutaminase co-activity. It is therefore desirable to limit this activity. Nguyen et al. created L-ASNase variants with ultra-low glutaminase co-activity by adapting specific amino acids in the active site to increase specificity towards Asn. *Era-TM2*, an *Erwinia chrysanthemi* A31I/E63Q/S254Q triple mutant, proved to be strongly efficacious both in vitro and in vivo against T-ALL and B-ALL, with reduced side effects [40,177]. Site-directed mutagenesis was also employed by Offman et al., who created a N24A/Y250L *E. coli* L-ASNase mutant with decreased glutaminase activity [178]. Immunogenicity on the other hand can be reduced by identifying and modifying antigenic residues in the enzyme. Jianhua et al. reduced antigenicity in *E. coli* L-ASNase through R195A/K1961/H197A mutations [179].

An alternative strategy to enzyme engineering is bioprospecting other microbial sources for L-ASNases with improved characteristics. In particular, extremophilic bacteria are an interesting novel L-ASNase source due to their superior pharmacokinetic properties. Therapeutics derived from halophilic bacteria often showcase increased biological activity and tolerance to the osmolarity of blood. Ghasemi et al. isolated L-ASNase from *Halomonas elongata*, which showed cytotoxicity in vitro, ameliorated half-life, and optimal activity at a temperature of 37 °C [180]. In the food industry, where L-ASNases are used for the reduction of carcinogenic acrylamide formation when frying food, thermophilic bacteria are used as a L-ASNase source, as these have increased thermostability [9]. This increased stability can also greatly benefit pharmaceutical applications. However, at physiological temperatures their activity is often greatly reduced. Research therefore is focused on generating specific mutations that preserve stability but ameliorate activity at these lower temperatures. Recently, a K274E mutant of *Pyrococcus furiosus* L-ASNases was created that preserved thermostability and greatly increased efficacy in vitro against human cell lines [181].

## 6. Combination Therapies

Identification of L-ASNase resistance mechanisms has fueled research into promising novel drug combinations. For example, the GCN2-ATF4 axis can be responsible for reinduction of *ASNS* upon AA starvation by L-ASNase in specific tumor cell lines [122,182,183] (Figure 1D). Of note, this L-ASNase resistance mechanism can be counteracted by using recently developed GCN2 inhibitors. Indeed, this combination therapy was shown to be highly efficient in the context of ALL, AML, pancreatic cancer, and melanoma [184,185,186]. Similarly, a recent study showed that the BTK inhibitor Ibrutinib strongly synergized with L-ASNase in ALL through a comparable mechanism that involved suppression of GCN2 activity [187]. In addition, it was shown that SLC1A3, an aspartate/glutamate transporter, contributes to L-ASNase resistance in PC3 prostate cancer cells and plays an important role in tumor initiation and progression in a mouse model for breast cancer metastasis [188]. These findings suggest that restrictive aspartate and glutamate uptake might improve L-ASNase efficacy in solid tumors.

Finally, recent studies revealed that upstream activation of the Wnt pathway can induce L-ASNase sensitivity in drug resistant ALL cells. Indeed, Wnt activation inhibits GSK3a-dependent protein ubiquitination and degradation, resulting in decreased Asn recycling and higher susceptibility towards L-ASNase treatment (Figure 1D). Notably, pharmacological inhibition of GSK3a was sufficient to mimic this effect in vivo using leukemia mouse models [189]. Interestingly, the same concept was also shown to be applicable for APC or β-catenin-mutant CRC [113], suggesting that GSK3a inhibition might act as a more general mechanism to sensitize a variety of tumor types towards L-ASNase therapy.

## 7. Conclusions

In summary, L-ASNase therapy has been proven to be a successful anticancer therapy, but most of the L-ASNase preparations currently used elicit undesirable and potentially treatment-limiting side effects. The development of improved microbial L-ASNases is a challenge due to their immunogenicity resulting in subsequent failure to achieve sufficient long-term L-ASNase activity.

In addition, studies show that L-ASNase may also have clinical potential for the treatment of solid cancers with low or no ASNS expression. However, because of the tolerability and toxicity issues, which are especially pronounced in adults, the clinical potential of L-ASNase for the treatment of these solid tumors has not fully been pursued. Therefore, one could speculate that the development of safer, less toxic, and non-immunogenic L-ASNase variants could expand its use to many other aggressive types of cancer with poor outcomes, including glioblastoma, breast, pancreas, and hepatocellular carcinoma. However, the role of glutaminase co-activity for the anticancer activity of L-ASNase is still a matter of debate, as literature shows conflicting reports. Some cancers may be dependent on Gln for their growth or become Gln dependent after Asn depletion and eventually will not benefit from alternative low glutaminase L-ASNase variants. These questions will have to be addressed to assess the therapeutic potential of alternative L-ASNases in clinical development. Further, characterization of L-ASNase resistance mechanisms provide a piece of the puzzle for further development of more personalized combination therapies, not only for ALL but also for solid cancer subtypes.

## Figures and Tables

**Table 1:** Overview of L-ASNase variants currently used in the clinic. This table shows a comparison of different L-ASNase variants, their production hosts, and potential tags. In addition, remarks concerning the approval of the product and findings as observed in literature are shown.

Name	Origin	Additions/Tags	Remarks	References
Elspar^®^	*E. coli* Type 2	Native		[35]
Kidrolase^®^	*E. coli* Type 2	Native		[36]
Spectrila^®^	*E. coli* Type 2	Native		[37]
Oncaspar^®^	*E. coli* Type 2	PEG (Succinimidyl succinate linker)		[25]
Asparlas^®^	*E. coli* Type 2	PEG (Succinimidyl carbonate linker)	Only FDA approved	[27] [28]
Erwinase^®^	*E. chrysanthemi* ansB gene	Native		[23] [24]
Rylaze^®^	*E. chrysanthemi* ansB gene	Native	Only FDA approved	[23] [24]

**Table 2:** Overview of solid tumors with potential to benefit from L-ASNase treatment. This table shows a comparison of different cancer types and their correlating L-ASNase sensitivity mechanism. In addition, possible combination therapies and findings as observed in literature are shown.

Cancer Type	L-ASNase Sensitivity Mechanism	Combination Therapy	Findings	References
Acute myeloid leukemia	ASNS-low	Methotrexate	Gln bio-availability is important for growth. Importance glutaminase co-activity is unclear	[106] [107] [108] [109]
		Cytarabine		
Ovarian clear cell carcinoma	ASNS-low		Glutaminase co-activity might be important for cytotoxic effect	[18] [110]
Pancreatic ductal adeno-carcinoma (PDAC)	ASNS-low		52% of PDAC classified as ASNS-low	[17]
	KRAS-mutated		Gln and Asn dependencies often observed	[111]
Colorectal cancer (CRC)	KRAS-mutated	Rapamycin	Suppression KRAS-mutated CRC	[112]
	WNT-mutated		Decreased Asn recycling increases susceptibility to L-ASNase	[113]
Metastatic breast cancer			Asn bioavailability governs metastatic potential	[20]
Hepatocellular carcinoma	ASNS-low		Low ASNS associated with worse prognosis, increased invasion, and metastatic potential but increased sensitivity to L-ASNase	[114]
	*ASNS* promoter methylation		Hypermethylation inhibits acquisition of resistance to L-ASNase	[115]
Glioblastoma		Temozolomide	Potential synergistic effect in vivo. Not confirmed yet if due to Asn/Gln depletion	[116]
		6-Diazo-5-oxo-l-norleucine	Synergistic anti-proliferative effect in glioma	[117]
